# 670. Precision Metagenomic (PM) Sequencing Outperforms Conventional Urine Culture in Detecting Clinically Relevant Microorganisms

**DOI:** 10.1093/ofid/ofab466.867

**Published:** 2021-12-04

**Authors:** Rita C Stinnett, Bethany Kent, Marta Mangifesta, Anagha Kadam, Heng Xie, Stacie Stauffer, Jamie Lemon, Benjamin Briggs, Lauge Farnaes, Robert Schlaberg

**Affiliations:** 1 IDbyDNA, Salt Lake City, Utah; 2 PathGroup Labs, Nashville, Tennessee

## Abstract

**Background:**

Morbidity from urinary tract infection (UTI) is high. Urine culture is the reference method for UTI diagnosis. Its diagnostic yield is limited as prior antibiotic use prevents growth of established uropathogens, many emerging uropathogens do not grow under routine culture conditions, and results interpretation can be subjective. Faster, more comprehensive diagnostics could help manage recurrent and/or drug-resistant infections. We evaluated the diagnostic yield of a precision metagenomic (PM) workflow for pathogen detection & antimicrobial resistance (AMR) characterization directly from urine.

**Methods:**

Residual urine samples from symptomatic adults evaluated by culture & susceptibility were identified by a combination of consecutive & stratified random sampling (n=480; 79% culture positive). DNA was extracted with modifications to the Quick-DNA Urine Kit (Zymo). Libraries were generated with Illumina DNA Prep with Enrichment for clinically relevant targets (191 pathogens, 1976 AMR markers) with the Explify Urinary ID/AMR Panel (UPIP, IDbyDNA). Enriched libraries were sequenced on the NextSeq550 (Illumina) and data analyzed with the Explify UPIP Data Analysis Solution (IDbyDNA).

**Results:**

For bacterial uropathogens, 94% positive agreement was observed between this PM workflow and culture. PM detected fastidious and/or anaerobic potential uropathogens in 30% and 7% of samples reported as culture-negative or positive for other bacteria, respectively. Total agreement between AMR marker detection and phenotypic resistance was 78%. Notably, PM predicted phenotypes of ESBL *E. coli* and *K. pneumoniae* (10/10), MRSA (9/9), and vancomycin-resistant *E. faecium* (4/5). PM also detected pathogens associated with sexually-transmitted infection (*C. trachomatis*, HSV) and bacterial vaginosis (*G. vaginalis*). PM produced complete results within 24-36 hours of sample receipt (vs culture & susceptibility: 42-72 hrs).

**Conclusion:**

The sensitivity of PM for uropathogen detection was noninferior to culture (Δ = 0.05; Nam RMLE; p < 0.0005). PM predicted antimicrobial resistance phenotypes for common uropathogens and identified potential pathogens not detected by conventional culture. Future studies should assess the impact of PM-guided management on clinical outcomes.

**Disclosures:**

**Rita C. Stinnett, PhD, MHS**, **IDbyDNA** (Employee) **Marta Mangifesta, PhD**, **IDbyDNA** (Employee) **Anagha Kadam, PhD**, **IDbyDNA** (Employee) **Heng Xie, PhD**, **IDbyDNA** (Employee) **Stacie Stauffer, BS**, **IDbyDNA** (Employee) **Jamie Lemon, PhD, D(ABMM**), **IDbyDNA** (Employee) **Benjamin Briggs, MD, PhD**, **IDbyDNA** (Employee) **Lauge Farnaes, MD, PhD**, **Cardea Bio** (Advisor or Review Panel member)**IDbyDNA** (Employee) **Robert Schlaberg, MD, MPH**, **IDbyDNA** (Consultant, Shareholder, Co-founder)

